# Using the UK standards for public involvement to evaluate the public involvement sections of annual reports from NIHR managed research centres

**DOI:** 10.1186/s40900-023-00517-3

**Published:** 2023-11-30

**Authors:** Alice Moult, Dereth Baker, Ali Aries, Paul Bailey, Steven Blackburn, Tom Kingstone, Saumu Lwembe, Zoe Paskins

**Affiliations:** 1 Impact Accelerator Unit, Keele University, Newcastle-under-Lyme, ST5 5BG UK; 2 School of Medicine, Keele University, Newcastle-under-Lyme, ST5 5BG UK; 3 School of Allied Health, Keele University, Newcastle-under-Lyme, ST5 5BG UK; 4 Patient and Public Involvement and Engagement, Institute of Applied Health Research, University of Birmingham, Edgbaston, Stoke on Trent, B15 2TT UK; 5 National Institute for Health and Care Research, LGC Ltd, Grange House, 15 Church St, Twickenham, TW1 3NL UK; 6 Haywood Academic Rheumatology Centre, Haywood Hospital, Midland Partnership University NHS Foundation Trust, Stoke on Trent, ST5 5BG UK

**Keywords:** Patient and Public Involvement and Engagement, Evaluation, Quality framework

## Abstract

**Background:**

Within the United Kingdom (UK), the National Institute for Health and Care Research is the largest funder of health and social care research, and additionally funds research centres that support the development and delivery of research. Each year, award-holders of these research centres are required to write a report about their activities, including a summary of Patient and Public Involvement and Engagement (PPIE) activities. This study aimed to evaluate the PPIE sections of annual reports to identify best practice and challenges; this could inform future delivery of PPIE activities.

**Methods:**

A framework documentary analysis informed by the six UK Standards for Public Involvement (‘Inclusive opportunities’, ‘Working together’, ‘Support and learning’, ‘Communications’, ‘Impact’ and ‘Governance’) was conducted on 112 reports. A quality improvement framework (‘Insights’) was used to evaluate quality as one of: ‘Welcoming’, ‘Listening’, ‘Learning’ and ‘Leading’. Recommendations from this review were co-developed with stakeholders and public contributors.

**Results:**

Reports documented varying levels of quality in PPIE activities which spanned across all six UK Standards. Award-holders either intended to, or were actively working towards, increasing access and inclusivity of public involvement opportunities. Methods of working with public contributors were varied, including virtual and in-person meetings. Most award-holders offered PPIE support and learning opportunities for both public contributors and staff. Some award-holders invited public contributors to co-produce communication plans relating to study materials and research findings. The impact of public involvement was described in terms of benefits to public contributors themselves, and on an organisation and project level. Many award-holders reported inviting public contributors to share decision-making within and about governance structures.

**Conclusions:**

This evaluation identified that most annual reports contained evidence of good quality PPIE practice with learning from public contributors. Using the UK Standards and Insights framework enabled exploration of the breadth and quality of PPIE activities. Recommendations include the need for a platform for centres to access and share PPIE best practice and for centres to collaborate with local and national partners to build relationships with the public through inclusive community engagement.

**Supplementary Information:**

The online version contains supplementary material available at 10.1186/s40900-023-00517-3.

## Abstract (plain language summary)

**What did we do?:**

Within the United Kingdom (UK) the National Institute for Health and Care Research (NIHR) is the largest funder of health and social care research. The NIHR also funds research centres that support the delivery of research studies. Each year, award-holders of these research centres are required to write a report describing their activities. These reports include activities related to Patient and Public Involvement and Engagement (PPIE). We aimed to evaluate the PPIE sections of these reports to identify best practice and challenges. This could, in turn, inform and aid researchers to enhance their PPIE approaches and improve how they work with the public in research.

**How did we do it?:**

We looked at 112 reports using the six UK Standards for Public Involvement (these include: ‘Inclusive opportunities’, ‘Working together’, ‘Support and learning’, ‘Communications’, ‘Impact’ and ‘Governance’). We used a quality improvement framework named ‘Insights’ to categorise PPIE practice into one of four levels of increasing quality: ‘Welcoming’, ‘Listening’, ‘Learning’ and ‘Leading’.

**What are the findings?:**

PPIE activities, of varying quality, covered all six UK Standards. A number of award-holders either intended, or were actively working towards, increasing access and inclusivity of public involvement opportunities. Methods of working with public contributors were varied. Most award-holders offered support and learning opportunities for both PPIE members and staff. Some award-holders invited PPIE members to co-produce communication plans relating to study materials and research findings. The impact of public involvement was described in terms of benefits to PPIE members themselves, and on a project and award-holder level. Many award-holders reported inviting public contributors to share decision-making within and about governance structures.

**What’s the bigger picture?:**

This evaluation identified that the Insights framework was useful in determining the quality of PPIE activities relating to each UK Standard. Recommendations for improving the quality of future PPIE activities were co-developed with staff from different research centres, senior leaders within the NIHR, PPIE leads and public contributors.

**Supplementary Information:**

The online version contains supplementary material available at 10.1186/s40900-023-00517-3.

## Background

Patient and public involvement can be defined as research carried out “with” or “by” patients and public contributors rather than “to”, “about” or “for” them [1]. Public engagement is the ways in which research can be shared with the public [2]. Patient and Public Involvement and Engagement (PPIE) is put into practice when researchers work with people who have lived experience of a thematic area of interest. It involves researchers working alongside public contributors to enhance study relevance, design, recruitment, data analysis, reporting and governance [3–5].

Within the United Kingdom (UK), the National Institute for Health and Care Research (NIHR) is the largest funding body for health and social care research. The NIHR provides funding for several research centres, such as Clinical Research Facilities (CRFs), Diagnostic Evidence Cooperatives (DECs) and Health Protection Research Units (HPRUs). These centres provide research expertise, specialist facilities, a research delivery workforce and support services which all help to facilitate the delivery of research funded by the NIHR and other stakeholders [6]. Award-holders (who conduct research supported by the research centres) are overseen by the NIHR Coordinating Centre. Research centres, and the award-holders which sit within them, are often at the forefront of supporting and promoting good practice in PPIE. For example, research centres and award-holders are active in engaging with local people and communities and show a commitment to developing new approaches for involving patients and the public within their own operations and activities [7].

The NIHR requires award-holders to produce an annual report describing the progress of their project against intended plans. Within these reports, and in relation to their PPIE strategies, award-holders are asked to provide a summary of their PPIE activities and, since 2019, describe how they are using the UK Standards for Public Involvement (referred to hereafter as the UK Standards) [8]. The UK Standards provide a framework for award-holders to express how they have involved and engaged with the public across six areas relating to: ‘Inclusive opportunities’, ‘Working together’, ‘Support and learning’, ‘Communications’, ‘Impact’ and ‘Governance’. The annual reports also provide award-holders with the opportunity to describe any challenges relating to PPIE activities. Building upon the recommendations from the NIHR’s 2015 strategic report for the future of public involvement, ‘Going the Extra Mile’ [9], the 2021 NIHR’s Improvement Plan [10] outlines a commitment to ensuring learning takes place from what does, and does not, work in practice. These annual reports give an opportunity for identifying and sharing good PPIE practice within and across research centres, but their content has not previously been formally evaluated.

**Fig. 1:**
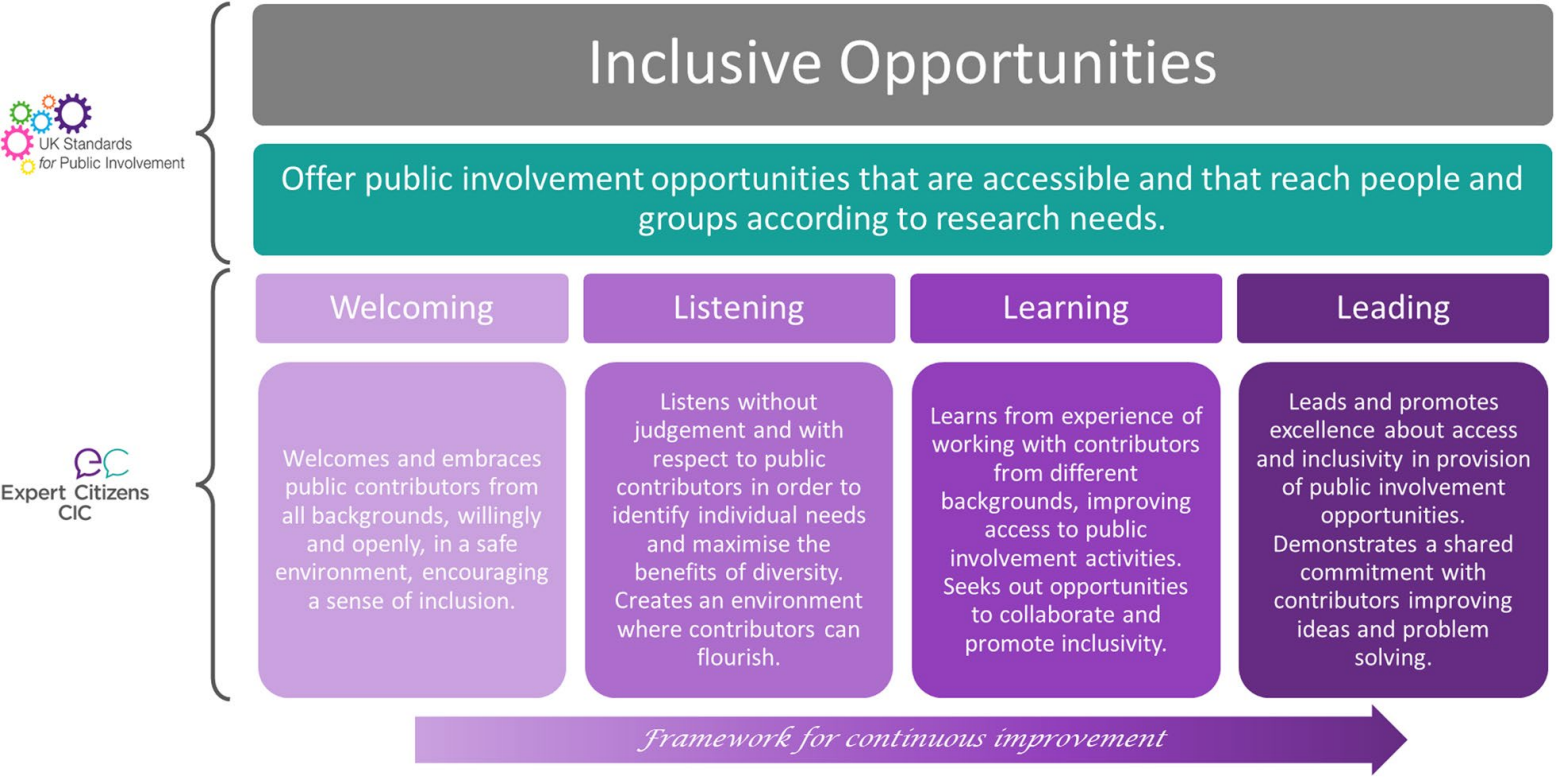
Example of the relationship between the UK Standards and the Insights framework for the inclusive opportunities standard

To develop learning, quality PPIE must be defined and shared. A quality improvement framework called the Insight | Public Involvement Quality Recognition and Awards Programme ([11], henceforth referred to as the Insight framework) has recently been developed as a partnership between Keele University and Expert Citizens Community Interest Company (a lived experience group based in Stoke-on-Trent; [12]). Insight categorises PPIE practice in relation to each of the six UK Standards in four levels of increasing quality: ‘Welcoming’ (provides welcoming, safe and supportive environments and offers a range of positive and inclusive involvement options and experiences), ‘Listening’ (listens actively to the views of people with lived experience and uses feedback to inform ways of working, services and systems), ‘Learning’ (continuously learns what works well for people and demonstrates that changes and improvements have been made from lived experience), and ‘Leading’ (leads the way and shares with others how people are involved in research and the benefits. Insights describes ‘Leading’ as ‘excellent’ PPIE practice, and ‘Learning’ as ‘good’ PPIE practice. Co-designing and/or co-producing an increasing number of aspects of research and encouraging public contributors as equal partners are embedded). The current utility of the Insight framework to evaluate PPIE activities is unknown. Figure 1 shows an example of the Insights framework and its relationship to the Inclusive Opportunities UK Standard.

The overall aim of this evaluation was to explore the breadth and quality of public involvement as reported by award-holders who sit within research centres. The objectives of this evaluation were: (1) evaluate the content of the PPIE sections of annual reports, to identify excellent and good PPIE practice and challenges reported by award-holders of research centres; and (2) to develop recommendations from the findings of this report for public contributors, researchers, senior research leads, award-holders and the NIHR.

## Methods

**Fig. 2:**
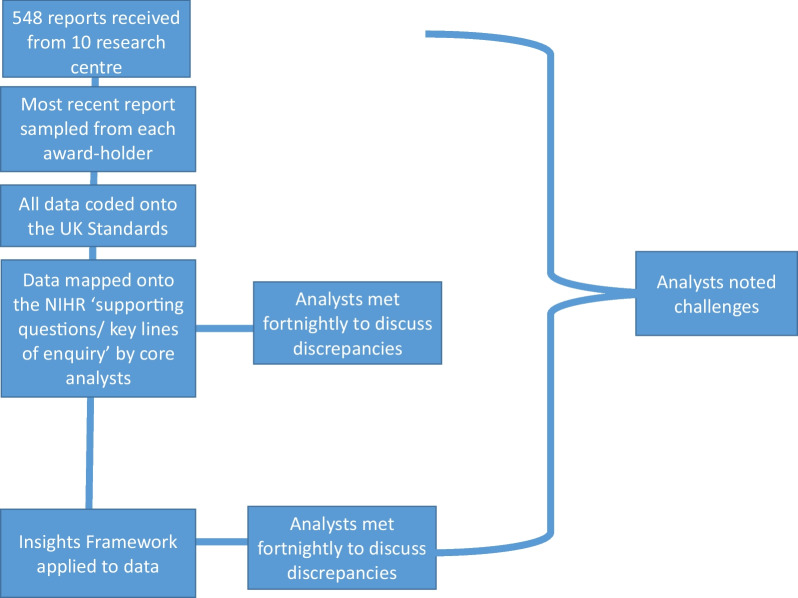
Flowchart of methods

A framework documentary analysis [13] of award-holders’ PPIE sections of annual reports, informed by the UK Standards and Insights framework, was conducted. The research team was led by Alice Moult (AM-Research Fellow in Knowledge Mobilisation). Other analysts included: Dereth Baker (DB-Research Assistant in Applied Health), Ali Aries (AA-Senior Lecturer in the School of Allied Health Professions), Tom Kingstone (TK-Lecturer in Mental Health and Wellbeing), Steven Blackburn (SB-Associate Professor in PPIE) and Zoe Paskins (ZP-Reader and Honorary Consultant in Rheumatology and applied health researcher). A flowchart describing the methods can be found within Fig. 2.

### Data source

With appropriate site approval from the award-holders within each of the 10 NIHR research centres, a total of 548 reports, produced between 2014 and 2021, were made available to the study team. The NIHR redacted any identifiable information before the reports were shared with Keele University. Keele University’s Research Ethics Committee (REC) advised that ethical approval was not needed for this study.

Table 1 details the different types of research centre, their research context, total number of reports submitted between 2014 and 2021 and total number of reports sampled within this evaluation. A glossary of each research centre can be found in Additional file 1.

**Table 1:** NIHR research centre information

Research centre	Research context (applied or experimental)	Total number of reports submitted between 2014 and 2021	Total number of reports sampled for this review
Biomedical Research Centres (BRCs) (n = 20)	Applied	110	20
Blood and Transplant Research Units (BTRUs) (n = 3)	Experimental	9	3
Collaborations for Leadership in Applied Health Research and Care/Applied Research Collaborations (CLAHRCs) (n = 15)	Applied	91	15
Clinical Research Facilities (CRFs) (n = 27)	Experimental	141	27
Diagnostic Evidence Cooperatives (DECs) (n = 4)	Applied	14	4
Health Protection Research Units (HPRUs) (n = 14)	Experimental	90	14
MedTech and In Vitro Diagnostics Co-operatives (MICs) (n = 11)	Experimental	33	11
Patient Safety Translational Research Centres (PSTRCs) (n = 3)	Applied	18	3
Research Design Service (RDS) (n = 10)	Applied	30	10
Research Schools (RS) (n = 5)	Applied	12	5
Total		548	112

### Sampling

To form an overall impression of the data, AM, DB and AA began by reading a random selection of 20 reports from different award-holders and years of submission. Researchers noted that the more recent reports contained more detail and more examples of best practice. After this, a subset of the 548 reports was selected for analysis to inform feasibility of the approach, whilst maintaining rigour and spread. Whilst there is variation between the number of reports submitted from each NIHR Research Centre, given the 2019 requirement for award-holders to include how they have used the UK Standards in the annual reports, the most recent report was selected from each award-holder to ensure the greatest likelihood that reports provided details in relation to the UK Standards.

### Data analysis

The process of analysis followed a modified version of the framework method [8] and included: developing and applying an initial deductive framework informed by the UK Standards and ‘supporting reflective questions’, developing the framework further with inductive themes, applying the Insights framework to describe quality and interpreting the data to identify challenges.

The UK Standards formed the first draft of the analytical framework, creating a structure to organise and summarise the data to answer the research questions. Using NVivo 12, AM, DB, AA, TK, ZP and SB independently extracted data line by line from the same five reports from five different award-holders and coded the data onto the six UK Standards. Each data extract could be coded against more than one UK Standard if deemed appropriate. The team met and discussed any discrepancies until a consensus was reached. A further 107 reports were split between three core analysts (AM, DB, AA) for data extraction. The three analysts recorded analytical notes, thoughts, or impressions of the data throughout and met fortnightly to discuss and resolve any queries which arose. The framework was iteratively amended as the research team developed ‘working definitions’ of the six UK Standards. These were developed and modified over the course of analysis based upon common illustrative phrases used by the award-holders, discussions between the research team, and from consulting the study’s PPIE group. The ‘working definitions’ aided extraction of all pertinent data related to the research question. Table 2 provides the original definitions of the UK Standards and the ‘working definitions’ used by the research team. Illustrative exemplars of modifications are underlined within the working definition.

**Table 2:**
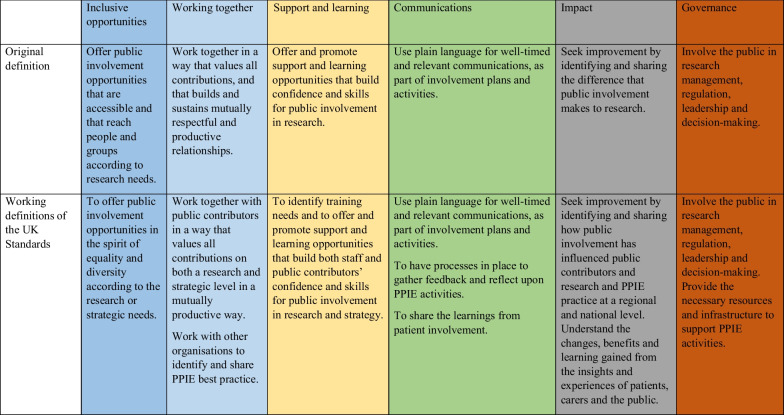
Definitions of the UK standards for public involvement

The next phase of developing the framework was to consider the data coded to each UK standard; this was to identify examples of the UK Standards in practice. The data was further deductively mapped to the ‘supporting reflective questions’ provided by the NIHR for each UK Standard [8].

The NIHR suggested that the supporting questions were developed to help researchers to reflect and decide if they have met the standard. Within the standard ‘Inclusive Opportunities’, an example of a supporting question is: ‘Are people affected by and interested in the research involved from the earliest stages?’ The framework integrated each of the ‘supporting reflective questions’ relating to each UK Standard. Thirdly, the data was further organised to identify themes within each ‘supporting reflective question’. An extract from the Data analysis framework can be found within Additional file 2.

Finally, to identify good PPIE practice, the Insights framework was used to determine the quality of PPIE activities within each UK Standard. AM, DB and AA applied the Insights framework to coded data within the framework, with one-rater coding each data extract. For each UK Standard the number of data excerpts coded as ‘Welcoming’, ‘Listening’, ‘Learning’ and ‘Leading’ were counted.

Throughout the research process the core research analysts noted any impressions, ideas and interpretations of data relating to any of the challenges that award-holders faced in delivering their PPIE activities (Table 3).

**Table 3:** The domains of the Insights framework coded to each UK Standard

UK Standards	Domains of the insights framework			
	Welcoming	Listening	Learning	Leading
Inclusive Opportunities	169	90	83	38
Working together	249	196	194	95
Support and Learning	142	191	148	103
Communications	107	104	110	47
Impact	81	69	66	40
Governance	41	37	32	19
Total number of excerpts:	689	687	642	336

### Stakeholder involvement

The researchers convened a PPIE group (two members of Keele Medical School’s Research User Group and one public contributor aligned to the School of Allied Health Professions at Keele University) specifically for this study. Whilst all three were invited to be a co-author on this paper, PB was the only member who accepted the invitation.

The aim was to integrate PPIE input into four key stages of the research process when developing the research questions, methods, and recommendations, and when interpreting the findings. Researchers used the GRIPP 2 framework to detail PPIE activities (please see Additional file 3).

Recommendations to improve the quality (e.g. to move from ‘welcoming’ to ‘leading’) of future PPIE activities were first developed between the PPIE group described above and research team but were further refined within a stakeholder event held on the 7th February 2023. A total of 73 stakeholders including staff from a range of different research centres, senior leaders within the NIHR, PPIE leads. The three members of the PPIE group attended the stakeholder event. Other PPIE members from Keele University’s Research User Group were invited, but only one further person attended the event.

The academic researchers approached all contact with PPIE members in an ethical manner. The researchers made it clear that the PPIE member is under no obligation to take part in any element of the project, and could leave the session at any time, providing written information about the project, nature of the activity and contact details of AM and encouraged PPIE members to discuss their involvement with their peers.

## Results

A total of 112 reports were analysed. Ten percent of the reports (n = 11) were submitted to the NIHR between 2016 and 2019, all other reports (n = 101) were dated 2019 and beyond. The majority of the reports which were published after the introduction of the UK Standards described PPIE activities which spanned across all six standards. A few of these reports used the UK Standards to structure the content of their report. Reports that were submitted prior to 2019 had less evidence of promoting inclusive opportunities, for example, including public members within governance structures, and capturing the impact of PPIE activities.

Most research centres had award-holders within them that reported examples of good and excellent PPIE activities (either coded as ‘Leading’ or ‘Learning’). In some reports submitted by award-holders hosted in experimental based research centres (e.g., Blood and Transplant Research Units; BTRUs) the technical subject matter was deemed a barrier to involvement. No other differences between experimental and applied research contexts were noted.

Fifteen individual themes relating to the six UK Standards were identified. Themes are identified in the text in italics. Illustrative quotes for each theme are shown in Table 4 and are numbered within the text (E.g., Q1 corresponds to Quote One in the table).

**Table 4:** Identified themes mapped to the UK Standards for Public Involvement

UK standards	Themes	Quotes	Insights Framework domain
Inclusive Opportunities	Widen involvement	Quote 1 (Q1): “We are working with groups across the nine protected characteristics covered by the Equality Act 2010 (age, disability, gender reassignment, marriage and civil partnership, pregnancy and maternity, race, religion or belief, sex, and sexual orientation). Identifying partners who work with vulnerable, minority and seldom heard populations is key to building mutually beneficial relationships and ensuring that a diverse, broad range of the population is represented by our research.” (MedTech and In Vitro Diagnostics Co-operatives; MICs)	Leading
	Strategic level	Quote 2 (Q2): “We worked in partnership with both long-standing and new PPIE contributors from under-served communities (including Lesbian, Gay Bisexual and Transgender (LGBT+), ethnic minorities, carers and young adults) to inform our BRC-4 PPIE Strategy.” (Biomedical Research Centre; BRC)	Learning
	Building sustainable relationships	Quote 3 (Q3):“We will co-produce a way forward for the partnership and identify what needs to change at local and national levels to ensure an ongoing relationship.” (Research Design School; RDS)	Leading
		Quote 4 (Q4): “We will continue to form new connections, sustaining community connections over time, or developing new ways of communicating and engaging with public members about research.” (BRC)	Learning
		Quote 5 (Q5): “Public engagement virtual event with (organisation). This organisation offers training and support for under-represented and ‘hard to reach’ minority ethnic groups, as well as economically and socially disadvantaged communities.” (MICs)	Welcoming
Working together	Defining public involvement	Quote 6 (Q6): “Series of strategy development workshops held where mixed groups of PPI representatives, academics, PhD students and PPIE leads from the region shaped draft objectives and discussed methods to deliver these. PPI representatives reviewed and shaped eventual PPI strategy document.” (Collaborations for Leadership in Applied Health Research and Care; CLAHRC)	Leading
	Methods of working together	Quote 7 (Q7): “Our PPIE Team organised and delivered the Summer School in June 2020; the school was delivered online to allow PPIE members to take part in a mixture of live interactive lessons, pre-recorded videos and group work in their own time.” (BRC)	Learning
	Stages of the research cycle	Quote 8 (Q8): “Members of the (PPIE) network have contributed to the production of a successful proposal to the NIHR PHRP for funding.” (Research School)	Learning
		Quote 9 (Q9): “Two public contributors are part of the study team. Both assisted with the refinement of the study design, developing patient-facing materials and have taken part in data analysis sessions to assist with interpretation of the findings.” (Research School)	Learning
		Quote 10 (Q10): “The study is a mental health service user-led project with over half the research team identifying as service user or survivor researchers, including the Principal Investigator, and two practitioner researchers.” (Research School)	Leading
	Collaborating with wider networks	Quote 11 (Q11): “We work with partners across the (geographical location) to share resources, identify joint projects and areas for collaboration, build capacity, develop community partnerships and promote best practice.” (BRC)	Leading
Support and Learning	Resources for PPIE members	Quote 12 (Q12): “The Involvement Coordinator continued to share links to resources and learning opportunities through a network of theme-based involvement champions and provide one-to-one support as required.” (Applied Research Centre)	Learning
		Quote 13 (Q13): “…Used ‘buddy’ system—had phone call with experienced public member prior to meeting to discuss how meetings worked, and to put at ease.” (ARC)	Welcoming
	Resources for academic staff	Quote 14 (Q14): “We delivered the first of a series of training sessions. Eight researchers attended and interactive polls pre and post training showed increased knowledge of PPI and increased confidence delivering PPI activities. PPIE members are also invited to attend training sessions.” (Patient Safety Translational Research Centres; PTSRC)	Learning
Communications	Co-producing study materials	Quote 15 (Q15) “(PPIE activity) included ensuring our research materials are easily understood i.e. PPI contributors helped write consent forms.” (Research School)	Learning
	Dissemination strategies	Quote 16 (Q16): “Most recently, we have seen the launch of videos involving LGBTQI + Disable People on the findings from (programme of studies) all of which have included relevant individuals within those videos and in developing the content for them.” (Research School)	Learning
	Digital inclusion	Quote 17 (Q17): “Researchers will aim to be flexible in offering virtual meetings and in providing a contribution towards telephone or broadband costs for virtual meetings. Where possible the meeting platform will enable people to dial in.” (Health Protection Research Unit; HPRU)	Learning
		Quote 18 (Q18): “…We offered technical support where necessary, (i.e., guidance on using online meeting platforms e.g., Zoom/ Microsoft teams). Ensured online meetings are not too long, with built in times for informal discussion or breaks.” (PTSRC)	Learning
		Quote 19: “Future work must offer both online and in-person options particularly as our activity now involves people nationally but also in response to positive contributor feedback that it is more accessible.” (BRC)	Learning
Impact	Levels of impact	Quote 20 (Q20): “Making the video—and a patient being the 'face' of the training—was an enjoyable and rewarding experience. I felt like an equal in the process and felt valued and respected in terms of my input.” (PPIE Member—BRC)	Leading
	Evaluating PPIE activities	Quote 21 (Q21): “Evaluating the impact of our PPI/E is a process of: Evaluating the activity against its aims to ensure the aims have been reached; Gathering feedback from participants involved in PPI/E both participant and researcher to have a clear pathway for future improvement and shared learning” (ARC)	Learning
Governance	Share decision making	Quote 22(Q22): “Public Involvement governance processes, leadership and a central budget are in place with representatives for all members ensuring collective ownership.” (Research School)	Leading

### Inclusive opportunities

The majority of award-holders described their intentions to *Widen Involvement* from under-served communities including: ethnic minorities, young people, older adults, working age individuals, patients with a variety of lived conditions, all genders, Lesbian, Gay, Bisexual, and Transgender (LGBT), Voluntary Sector, Faith Communities and individuals with learning difficulties (Q1). Several award-holders described that they were ‘working with’ under-served communities within research projects but did not explicitly document how they involved individuals from such communities.

Most award-holders reported offering inclusive PPIE activities at a *Strategic Level* (Q2). Inclusive PPIE activities were not only integrated into strategic decision-making on a project level, but also at the research centre level through the planning and prioritisation of research programmes. Again, most reports did not include details of how public contributors were involved in such activities.

Several award-holders described their intention and commitment to *Building and Sustaining Relationships* with under-served groups (Q3). Despite there being a recognition from most award-holders that this would take time, and would need appropriate resourcing (e.g., staff), award-holders were committed and discussed adapting their communication strategies to meet the needs of under-served communities (Q4). Award-holders also recognised that a challenge when building sustainable relationships with under-served communities may be the additional support required (Q5).

### Working together

Some award-holders described working with public contributors when *Defining Public Involvement* through the co-production of frameworks and PPIE strategies (Q6). These award-holders reported that it was important that the co-produced frameworks and strategies worked for not only the public contributors, but all stakeholders within the research.

The *Methods of Working Together* were discussed in most reports. Different ways of working together included: in-person meetings, virtual group activities, telephone conversations and online or postal surveys. The methods of working together were often based upon practical requirements and needs of public contributors e.g., providing activities that public contributors could complete asynchronously at a time that best suited them due to work or education commitments (Q7).

A majority of reports described how researchers worked with public contributors at all *Stages of the Research Cycle*, examples included: identifying research priorities, co-producing and collaborating on grant applications and co-designing research methods influencing the research design, study recruitment, data collection and analysis (Q8; Q9). A small number of award-holders reported that public contributors conducted their own user-led research project (Q10).

In addition to working with public contributors, many of the reports provided examples of award-holders *Collaborating with Wider Networks* (research groups, third sector organisations and charities). Several award-holders described working with other award-holders to share resources and promote best practice (Q11).

Some award-holders described the challenge of keeping pace with local NHS restructuring. Due to this, award-holders expressed the need for increased collaboration across the NHS and other organisations. Some award-holders suggested that pooling PPIE resources would be beneficial for greater impact and consistency.

### Support and learning

Most award-holders provided *Resources for Public Contributors*, for example*,* award-holders with a designated PPIE co-ordinator provided access to knowledge about a public contributor’s role, along with any learning opportunities available through the research centre, through this individual (Q12). Other award-holders offered ‘taster sessions’ via video, peer support (Q13) and training for a specific project. The support offered to public contributors built their skills, knowledge, and confidence to contribute within PPIE activities. Training for public contributors involved in research governance activities (e.g., data and project management) was not described.

Most award-holders also provided *Resources for Academic Staff*. These materials were often co-produced with public contributors and included workshops, educational sessions (Q14) and PPIE clinics where researchers could get advice on their PPIE plans from specialist academics and public contributors. A few award-holders reported that researchers wrote PPIE-focused blogs targeted at their peers and one Research School identified that researchers needed help when planning PPIE activities which resulted in them developing a PPIE toolkit.

### Communications

Most award-holders described *Co-producing Study Materials* with public contributors (e.g. consent forms and participant information sheets). The award-holders that did this suggested that PPIE input ensured that the wording of such documents was appropriate for the target audience (Q15).

Several award-holders described that public contributors developed dissemination strategies for a project’s findings within their local community. Public contributors generally suggested more innovative and creative methods of communicating research findings. Such methods included illustrations, videos and graphic novels (Q16).

Following the response to the Covid-19 pandemic, award-holders described the rapid move to digital PPIE activities and the need to ensure *Digital Inclusion* for all public contributors. Despite digital inclusion being identified as the most prominent challenge described within the reports, most award-holders showed a great deal of innovation and reported advantages of digital PPIE activities such as the widening of PPIE involvement through the inclusion of people with mobility issues. To foster digital inclusion, award-holders reported providing access to technology, technical support, and financial resources (Q17; Q18). Looking to the future, a hybrid model could suit public contributors’ needs and offer a choice of how to be involved either digitally or in-person (Q19).

### Impact

Award-holders reported various *Levels of Impact.* The impact of PPIE activities upon the public contributors themselves was described (Q20), along with the impact of PPIE activities upon research projects and programmes. Some reports described the impact of PPIE activities at both regional and national levels e.g., co-produced terms of references being used within regional involvement networks and by national research councils.

Many award-holders described *Evaluating PPIE Activities* (Q21). Whilst a few award-holders co-produced frameworks with public contributors, most award-holders reported using frameworks such as the Public Involvement Impact Assessment Framework (PiiAF; [14]) Key Performance Indicators (KPIs) and ‘The cube’ [15] to assess impact. Following the evaluation of public involvement, some award-holders communicated changes to their PPIE practices to public contributors.

### Governance

Many award-holders reported inviting public contributors to *Share Decision-Making* within and about governance structures, including financial decisions (Q22). Public contributors were invited to join award-holder committees or board meetings where existing PPIE structures were reviewed and monitored. Overall, public voices were heard, valued, and respected on a project, programme, organisational and regional level.

### PPIE quality

In relation to the Insights framework, the domains ‘Welcoming’, ‘Listening’ and ‘Learning’ were strongly demonstrated throughout the reports (with 689, 687 and 642 data excerpts respectively coded to these domains). Leading PPIE activities were not as prevalent (336 excerpts). Table 3 illustrates the number of times each of domain of the Insights framework was coded to each UK Standard.

### Challenges in PPIE

Notably, only a small number of reports detailed any challenges in relation to the delivery of PPIE activities. Due to the response to the Covid-19 pandemic and a rapid move to on-line working, the main challenge described was needing to rapidly learn how to digitally engage (e.g., to move from in-person PPIE activities to online). Another reported challenge was to keep pace with the local National Health Service (NHS) restructuring (e.g., where Acute Trusts have amalgamated into merged multi-hospital organisations across larger conurbations, and national commissioner arrangements change between specialist, tertiary and locally commissioned services) and the loss of staff due to this restructuring. Within the stakeholder event, additional challenges were reported such as: ensuring that studies conducted by commercial partners (who may sponsor an award-holder or research project) have good quality PPIE, a lack of support to share learning across award-holders and research centres and knowing how to best engage with under-served communities.

## Discussion

This evaluation used the UK Standards and the Insights framework to explore the breadth and quality of public involvement reported by NIHR research centres. Following the introduction of the UK Standards, and the NIHR encouraging award-holders to refer to the UK Standards within their annual reports, award-holders described a breadth of activities that spanned all six of the UK Standards. Previous research has suggested that the UK Standards provide a benchmark to which researchers should aim to achieve [16]. This study has highlighted that there is no single way of meeting each of the UK Standards. Most of the award-holders reported different ways in which the UK Standards were applied, and their use was influenced by contextual factors, such as the nature of the award-holder (applied or experimental), purpose of involvement, and the available resources (finances, staff such as a PPIE co-ordinators and time).

The definitions of each UK Standard were originally provided by the UK Standards Partnership in consultation with the UK research community and their wider networks [8]. The ‘working definitions’ shown in Table 2 highlight areas of where the original definitions of the UK standards could be made more specific to help researchers when using the framework to evaluate PPIE practice. The researchers, and public contributors, particularly thought that the ‘Communications’ standard needed to be expanded to include having processes to gather feedback and reflections and could also include the sharing of learnings from PPIE activities. The definitions of ‘Inclusive opportunities’ and ‘Working together’ could be expanded to include how these standards could be met on a strategic level.

It was further noted that some UK Standards related more to public involvement (being actively involved in research projects and research organisations), and do not explicitly refer to public engagement (where information and knowledge about research is shared with the public). Most of the award-holders reported activities relating to patient and public involvement; only a few award-holders described engagement activities (e.g. disseminating research findings within local communities). Due to the importance of engaging with people and communities to foster relationships for research and, more generally, to promote research participation and sustaining diverse and inclusive public involvement, the UK Standards could be broadened to include quality in engagement activities, which in turn may influence reporting in this context.

To the best of the research team’s knowledge, this is the first study to determine the quality of PPIE activities relating to each of the UK Standards [11]. By using the four domains proposed by the Insights framework as markers of quality, researchers could distinguish between PPIE activities within each UK Standard. The Insights framework does not give specific reference to any challenges faced when delivering PPIE activities relating to each quality domain; however, this information could be useful when looking to improve the quality of PPIE activities.

The foundational domains of ‘Welcoming’ and ‘Listening’ were prevalent across all six of the UK Standards. The domains ‘Leading’ and ‘Learning’ were most prevalent with the ‘Support and learning’ and ‘Working together’ standards. The results show that the ‘Governance’ standard could be the most challenging area of PPIE to achieve the higher levels of quality. Further research is required to better understand this observation. Furthermore, the annual reports did not detail the data governance of public contributors’ personal information. While research centres are well versed to ensuring UK Data Privacy Requirements [17], specific guidance relating to public contributors’ data could be welcomed.

‘Leading’ and ‘learning’ PPIE practice was evidenced when award-holders described actual outcomes, rather than aims or ambitions, of their PPIE activities (e.g., the development of a co-produced toolkit for researchers or a public contributor leading a research project). When describing intended PPIE activities, award-holders particularly reported ambitions to widen involvement from under-served communities. Clearly, the time taken to build and foster sustainable relationships with these communities remains a challenge and not necessarily conducive to an annual reporting cycle. To move from intentions to having outcomes relating to widening involvement, award-holders may need adequate resources (e.g., staff who conduct outreach work within specific communities) to build these relationships. Award-holders may also benefit from other award-holders sharing best practice and the NIHR signposting to existing guidance (e.g., the NIHR Race Equality Framework; [18]). Whilst the Race Equality Framework focuses on providing a voice to those from Black, African, Asian or Caribbean heritage within a research environment, there may be generalised learning across wider under-served communities [18].

Whilst the most prominent challenge reported by award-holders was the need to rapidly learn how to digitally engage due to the restrictions put into place during the Covid-19 pandemic, most award-holders described adapting to a new model for remote PPIE. Looking to the future, a hybrid working model which involves both online and in-person PPIE activities may meet the needs of many PPIE members and increase accessibility [19]. A hybrid working model must enable equity in experience between those involved and ensure that people who wish to join online are supported to do so. A further challenge was local NHS restructuring and loss of resources. Due to this, award-holders wished to work more collaboratively to share resources. Future research is needed to inform, help and incentivise institutions to work together and pool resources.

### Limitations

This evaluation is subject to a number of limitations. In the main, data extraction and coding was completed by single analysts, although regular meetings ensured consistency in approaches. The Insights framework was also applied by single raters; we did not examine inter-rater reliability. The reports were assumed to be written from the perspective of each award-holder within a research centre. It is not known how involved PPIE leads (those who co-ordinate PPIE activities) or public contributors were in the development and writing of individual reports, and whether bias may have been present. Understanding who completed the reports, and the role of public contributors, would be of further benefit. Furthermore, the longer-term impact of public involvement in research studies, governance/operations of an award-holder, and/or relationships with communities are not realised within a single reporting year, therefore, taking a longer-term lens may be needed.

Finally, whilst the annual reports have shown examples of PPIE best practice, they might have provided a skewed interpretation of activities. Ultimately the annual reports are a requirement of a funding body, therefore, accounts of PPIE activities may be described more positively than is the actual case. Introducing a degree of independent appraisal of intended and actual PPIE activities may help to eliminate this bias. An alternative approach would be to actively encourage reporting of challenges and difficulties.

### Recommendations

To inform future high-quality delivery of PPIE activities, recommendations were co-developed with stakeholders and public contributors and are offered for public contributors, researchers senior research leads and PPIE leads, award-holders and the NIHR. These recommendations are presented within Table 5.

**Table 5:** Recommendations

*For public contributors, researchers, PPIE leads, senior research leads and award holders:*	
1.	To continue to apply the UK Standards as a basis for good PPIE practice To co-produce effective and inclusive ways of working together which include both online and in-
2.	person PPIE activities
3.	To collaborate with local and national partners to build relationships with the public through inclusive community engagement, particularly with under-served communities
4.	To work with public contributors when reporting PPIE activities within the annual reports
5.	To report on the outcomes of PPIE, where appropriate, instead of ambitions or processes alone
6.	To encourage the reporting of the challenges and successes relating to PPIE activities
*For the NIHR (as a research centre funder)*	
7.	To create a platform which enables research centres to share and reward areas of PPIE excellence
8.	To co-produce a guide or ‘tool kit’ for gathering and managing public contributors’ information appropriately and securely
9.	To consider how commercial partners can be supported to increase the breadth and quality of PPIE activities
10.	To encourage and support the further adoption of the NIHR Race Equality Framework [16] and to provide further guidance regarding how best to involve people from other underserved communities and people with protected characteristics
11.	To share opportunities for public involvement across research centres to increase the diversity and reach of public involvement
12.	To promote the broadening of the UK Standards to include engagement activities

## Conclusion

This evaluation found that the majority of award-holders demonstrate good quality PPIE practice across the six UK Standards. The UK Standards and Insights framework were useful in identifying different levels and aspects of PPIE activities. Challenges to PPIE activities included needing to learn how to digitally engage, the time-consuming nature of building relationships with under-served communities and local NHS restructuring. Recommendations, co-developed with stakeholders and public contributors, are offered for public contributors, researchers and senior research leads, award-holders and the NIHR.

### Supplementary Information

**Additional file 1**. Glossary of Research Schemes.

**Additional file 2**. Extract of Data Analysis Framework.

**Additional file 3**. The GRIPP 2 Short Form.

## Data Availability

Data for this study will be made available to the scientific community on request after publication. Data will be made available for scientific purposes for researchers whose proposed use of the data has been approved by a publication committee. Data and documentation will be made available through a secure file exchange platform after approval of proposal and a data transfer agreement is signed (which defines obligations that the data requester must adhere to regarding privacy and data handling). For data access, please contact the corresponding author.

## Abbreviations

ARC: Applied Research Collaborations
BRCs: Biomedical Research Centres
BTRUs: Blood and Transplant Research Units
CLAHRCs: Collaborations for Leadership in Applied Health Research and Care
CRFs: Clinical Research Facilities
DECs: Diagnostic Evidence Cooperatives
HPRUs: Health Protection Research Units
MICs: MedTech and In Vitro Diagnostics Co-operatives
NHS: National Health Service
NIHR: The National Institute for Health and care Research
PSTRCs: Patient Safety Translational Research Centres
PPIE: Patient and Public Involvement and Engagement
RDS: Research Design Service
SPHR: School for Public Health Research
SPCR: School for Primary Care Research
SSCR: School for Social Care Research
