# Supplementary material for: Using the UK standards for public involvement to evaluate the public involvement sections of annual reports from NIHR managed research centres
Source: Res Involv Engagem. 2023 Nov 30;9:109. doi: 10.1186/s40900-023-00517-3 (PMC10688454; doi:10.1186/s40900-023-00517-3)
Supplement: Supplementary file 1 — Additional file 1. Glossary of Research Schemes. [file 40900_2023_517_MOESM1_ESM.docx]

Additional File One: Glossary of Research Schemes

| **Applied Research Collaboration** | The NIHR Applied Research Collaborations support applied health and care research that responds to, and meets, the needs of local populations and local health and care systems. |
| --- | --- |
| **Biomedical Research Centre** | Biomedical Research Centres (BRCs) are collaborations between world-leading universities and NHS organisations that bring together academics and clinicians to translate lab-based scientific breakthroughs into potential new treatments, diagnostics and medical technologies. |
| **Blood and Transplant Research Unit** | Blood and Transplant Research Units are aimed at providing new technologies, techniques or insights that will benefit donation, transfusion, and transplantation. Many of the work strands involved could result in new technologies and practices that can then be delivered at scale, helping to save and improve even more lives. Much of the work will also be aimed at reducing health disparities and improving access to new treatments. |
| **Collaborations for Leadership in Applied Health Research and Care** | Collaborations for Leadership in Applied Health Research and Care are a collaboration of academics, clinicians and managers who undertake high quality applied health research focused on the needs of patients and service users, supporting the translation of research evidence into practice in the NHS and social care. |
| **Clinical Research Facilities** | Clinical Research Facilities (CRFs) are purpose-built facilities in NHS hospitals where researchers can deliver early-phase and complex studies. The facilities have cutting-edge clinical facilities, technologies and expertise and are designed to support high intensity studies and overnight stays. Researchers funded by the NIHR, the life sciences industry or other organisations can access assistance from skilled CRF clinical trial support staff, from study design to data collection and study management. |
| **Diagnostic Evidence Cooperative** | The aim of Diagnostic Evidence Cooperatives is to facilitate the development and evaluation of clinically relevant in vitro diagnostics, by offering methodological expertise and access to real-life settings for evaluations in patients. |
| **Health Protection Research Unit** | Health Protection Research Units are research partnerships between universities and Public Health England (PHE). The units act as centres of excellence in multidisciplinary health protection research in England. |
| **MedTech and In Vitro Diagnostics Co-operatives** | MedTech and In Vitro Diagnostics Co-operatives aim to build expertise and capacity in the NHS to develop and evaluate new medical technologies and in vitro diagnostic tests. Each MIC has a different focus and is hosted by a different NHS trust. |
| **Patient Safety Translational Research Centres** | Patient Safety Translational Research Centres mission is to work with patients, carers and health & social care staff and providers to develop and test ways of doing things (interventions) to improve patient safety. |
| **Research Design Service** | The Research Design Service provides support on all aspects of developing and writing a funding application, including research design, research methods, identifying funding sources and involving patients and the public. |
| **School for Public Health Research** | Each national school is a unique collaboration between leading academic centres in England, carrying out outstanding research in their respective fields. This research can be applied across the country to meet the needs of policymakers, practitioners and the public. School for Public Health Research aims to increase the evidence base for cost-effective public health practice. |
| **School for Primary Care Research** | School for Primary Care Research is a partnership between nine leading academic centres for primary care research. We work to increase the evidence base for primary care practice through high quality research and strategic leadership and to build capacity in primary care with a well-established training programme. |
| **School for Social Care Research** | The School for Social Care Research is a partnership between the London School of Economics and Political Science, King’s College London and the Universities of Birmingham, Bristol, Kent, Manchester and York, and is funded by the National Institute for Health and Care Research. |
