# Supplementary material for: Using the UK standards for public involvement to evaluate the public involvement sections of annual reports from NIHR managed research centres
Source: Res Involv Engagem. 2023 Nov 30;9:109. doi: 10.1186/s40900-023-00517-3 (PMC10688454; doi:10.1186/s40900-023-00517-3)
Supplement: Supplementary file 2 — Additional file 2. Extract of Data Analysis Framework. [file 40900_2023_517_MOESM2_ESM.docx]

Additional File Two: Extract of Data Analysis Framework

| **UK Standards for public Involvement – Key Lines of Enquiry/ Supportive Questions** | | **Interpretation** | **Themes, Supporting Data and links to Quality Ratings Framework**  (Links to the Quality Ratings Framework are in brackets and red font after each quote) | |
| --- | --- | --- | --- | --- |
| **Inclusive opportunities** | | | | |
| Are people affected by and interested in the research involved from the earliest stages? | | PPIE inclusivity was reported at a strategic level e.g. planning and prioritisation of research programmes and/ or at the earliest stages of project design | **Strategy**  BRC: We worked in partnership with both long-standing and new PPIE contributors from under-served communities (including Lesbian, Gay Bisexual and Transgender (LGBT+), ethnic minorities, carers and young adults) to inform our BRC-4 PPIE Strategy.’ (Learning)  BRC: “Working with community leaders on methods of involvement for socially/economically deprived wards where levels of smoking, alcohol and drug misuse are significantly elevated. Outcomes have been incorporated in PPI/E Plans across the BRC portfolio.’ (Listening)  **Research:**  MICS: “Older Adults and people with Dementia are frequently under-represented in research, particularly in med tech development. We have designed and delivered the PPI involvement for the [project]. Older adults and people with dementia are supported to participate in online focus groups to discuss their views.” (Listening) | |
| Have barriers to involvement, such as payment for time or accessible locations for meetings been identified and addressed? | | Examples of reimbursement and use of digital platforms were evident | **Reimbursement**  MICS: “The NIHR Centre for Engagement & Dissemination Payment Policy has been cited as helpful to reimburse public contributors in line with this.’ (Welcoming)  **Digital platforms**  MICS: ‘public engagement virtual event with [organisation]. This organisation offers training and support for under-represented and ‘hard to reach’ minority ethnic groups, as well as economically and socially disadvantaged communities.’ (Welcoming) | |
| How is information about opportunities shared, and does it appeal to different communities? | | Centres provided opportunities to include the diversity of population including race, age-ranges, working, faith, sexuality, gender, culture, ethic minorities, deprived communities etc. | BRC: “We have successfully recruited 11 new members to the group, nine of whom are of working age and nine of whom are from the Black Asian and Minority Ethnic backgrounds. Fundamental to the recruitment of new members was adherence to the Inclusive Opportunities standard and ensuring that the new members reflected the communities of [geographical location]’ (Learning)  BRC: “We worked in partnership with both long-standing and new PPIE contributors from under-served communities (including Lesbian, Gay Bisexual and Transgender (LGBT+), ethnic minorities, carers and young adults) to inform our BRC-4 PPIE Strategy.’ (Leading)  BRC: *‘*And contributors from deprived neighbourhoods are involved, with about half of contributors living in the 30% most deprived neighbourhoods’ (Welcoming)  CRF: “While our geographical area is predominantly Caucasian (>98%), 6% of our research volunteer cohort identify as an ethnic minority. To increase this proportion further the RD&E BAME staff network has agreed to act as a research resource” (Learning) | |
| Are there fair and transparent processes for involving the public in research, and do they reflect equality and diversity duties? | | Most reports demonstrate huge and innovative efforts to widen participation and involvement in PPIE from diverse populations/ communities including BAME, young people, older age, working age, patients with lived conditions, all gender,(s) LGBT , Voluntary Sector Faith Communities (VSFC) etc.  Enabling diverse communities to access PPIE activities by using different mediums and conduits/ fora and language.  We looked for evidence of the ‘process’ with regard to EDI duties, however most reports described the ‘who’ rather than the ‘how’ with reference to reflecting equality and diversity duties.  Centres discussed hoping to forge sustainable relationships.  Some infrastructure schemes made explicit reference to the UK Standards and the INVOLVE Ethnicity Framework and across the nine protected characteristics covered by the Equality Act 2010 (age, disability, gender reassignment, marriage and civil partnership, pregnancy and maternity, race, religion or belief, sex, and sexual orientation) | **Equality and diversity**  BRC: “80 public contributors responded to the 2021 equality, diversity and inclusion survey” (Welcoming)  Research School: “Our team developed patient materials in eleven different languages used in the UK, including Arabic, Bengali, Chinese, French, Hindi, Polish, Portuguese, Punjabi, Somali, Urdu and Welsh.’[MICS  Our studies are working with young adult carers, people with behaviours that challenge, people with learning disabilities, people with mental health needs – among many others – and have provided inclusive opportunities for individuals from these groups to be involved in projects.’ (Learning)  MICS: “Supporting multi-research centre anti-racism initiative, with membership of the Institute of Mental Health’s ‘Black Lives Matter Research Group” (Learning)  MICS: “We are working with groups across the nine protected characteristics covered by the Equality Act 2010 (age, disability, gender reassignment, marriage and civil partnership, pregnancy and maternity, race, religion or belief, sex, and sexual orientation). Identifying partners who work with vulnerable, minority and seldom heard populations is key to building mutually beneficial relationships and ensuring that a diverse, broad range of the population is represented by our research’ (Leading)  **Sustainability**  RDS: “We will co-produce a way forward for the partnership and identify what needs to change at local and national levels to ensure an ongoing relationship” (Leading)  **Involve framework**  MICS: ‘We continue to be guided by the UK Quality Standards and the INVOLVE Ethnicity Framework. We have maintained linkages across NIHR and NHS infrastructure PPI/E/P networks.” (Welcoming) | |
| Is there choice and flexibility in opportunities offered to the public? | | There was flexibility in opportunities. Virtual and face to face meetings were reported (see Case Study on Digital Inclusion within the ‘Communication’ standard).  Infrastructure schemes are recognising that some individuals with lived experience are also constrained by their respective health issues, which inhibits them from becoming fully involved and/ or some themed projects have the same individuals being involved. | **Discussing choice**  BRC: ‘Contact was made with the voluntary, community, faith sector (VCFS) for organisations to participate in Q&A focus groups’ (Welcoming)  **Range of groups**  PTSRU: “Working with a diverse range of public contributors remotely (people experiencing homelessness, mental health problems, visual impairment, dementia, long-term health problems, BAME communities, and carers, as well as people shielding” (Learning) | |
| **UK Standards for public Involvement – Key Lines of Enquiry/ Supportive Questions** | | **Interpretation** | **Themes, Supporting Data and links to Quality Ratings Framework**  (Links to the Quality Ratings Framework are in brackets after each quote) | |
| **Working together** | | | | |
| Has the purpose of public involvement been jointly defined and recorded? | | Centres reported jointly defining PPIE activities through the co-production of frameworks and strategies. | **Defining Public involvement**  CLARC: ‘Identify and develop a framework for co-production that works for our public members and research themes and in which public members feel valued for their input’ (Leading)  CLARC: ‘Series of strategy development workshops held where mixed groups of PPI representatives, academics, PhD students and PPIE leads from region shaped draft objectives and discussed methods to deliver these. PPI representatives reviewed and shaped eventual [PPI strategy document](https://www.arc-nt.nihr.ac.uk/media/sszf3irq/nihr-arc-north-thames-ppi-strategy-31-03-2021.pdf). We used UK Standards for Public Involvement as framework for our objectives. Payment policies updated in line with NIHR CED.’ (Leading) | |
| Have the practical requirements and arrangements for working together been addressed? | | Centres offered different ways of working together including group activities, telephone communication and text-based methods.  The practical requirements were based on the needs of the PPIE members (e.g. delivery of activities online due to students being at school in the day).  Most reports were written during the Covid 19 pandemic where centre’s had to rapidly adjust to working virtually. Please see ‘Digital inclusion’ case study within the ‘Communication’ standard. | **Ways of working together**  BRC: “The core PPI team hosted 37 study-specific PPI/focus groups for 31 projects and the upcoming BRC competition. These were mainly online focus groups, some telephone interviews and text-based discourse/surveys. Only 5 studies started this year had no public involvement.” (Learning)  **Practicalities:**  BRC: “Our PPIE Team organised and delivered the Summer School in June 2020; the school was delivered online to allow (PPIE members) to take part in a mixture of live interactive lessons, pre-recorded videos and group work in their own time.” (Learning) | |
| Have all the potential different ways of working together been explored, and have these plans and activities been developed together?  Is there a shared understanding of roles, responsibilities and expectations of public involvement? | | PPIE members took on a variety of different roles and responsibilities on both a research and strategic level; but it was unclear how these roles and activities were developed.  Centres reported working together will PPIE members to: support user-led research, identify research priorities, generate proposals, collaborate on grant application and co-design research methods (including research protocols, design, interventions, recruitment and analysis).  There are many examples of broader collaborations across sectors within the research networks, and beyond into third sectors/ charities etc. e.g. vascular dementia theme group including representatives from the Alztheimers’ Society.  Centres also described working with other centre’s to share best practice, which often included PPIE input. | **User-led research**  RDS: “We are very proud of a public partner who has won a £1m NIHR award as a Co-Lead for a project inspired by her lived experience. We provided hands-on support and have reflected on the process in a paper which highlights some of the challenges and lessons learned.” (Leading)  Research school: “The study is a user-led project with over half the research team identifying as service user or survivor researchers, including the Principal Investigator, and two practitioner researchers.” (Leading)  **Identifying research priorities**  BRC: “Recruitment of Youth Champions to advocate for the voice of CYP in research, and events including ‘ideathons’ and ‘Dragons Den’ to set priorities for research.” (Learning)  **Generating proposals**  PSTRU: “The PPIE team and PSTRC researchers are working with [group] on a project to co-produce a peer support solution for young people bereaved by suicide, funded by [funding body]. A Young Person’s Advisory Group with lived experience has been set up, who has met twice to develop the proposal and will work with us using user-centred design approaches to come up with a solution, aiming to support young people and reduce suicide ideation.” (Learning)  **Grant applications**  Research school:“Members of the (PPIE) network have contributed to the production of a successful proposal to the NIHR PHRP for funding” (Learning)  BRC:  “(PPIE members) gave input to 11 studies/grant proposals” (Learning)  **Methods**  Research school: “(PPIE activity) included: making sure our research is relevant to (target population) (i.e. modifying our (intervention); ensuring our research materials are easily understood (i.e. PPI contributors helped write consent forms); making sure our research process is acceptable to potential participants (i.e. modifying research protocol).” (Learning)  Research school: “A source of expertise and/or appointed specialist public advisers to help with recruitment.” (Learning)  HPRU: “The public involvement fund has supported six bids, including the added value example which highlights how the fund enabled researchers to engage female veterans as active partners and involve them in shaping key aspects of the design.” (Learning)  Research School: “Two public contributors are part of the study team. Both assisted with the refinement of the study design, developing patient-facing materials and have taken part in data analysis sessions to assist with interpretation of the findings.” (Learning)  **Strategy**  Research School: “We have also developed a working group bringing together PPI, Knowledge Mobilisation, Comms and training to ensure the joined up development/ delivery of our strategic aims.” (Learning)  **Working together (centre level)**  BRC: “Sharing between centres We work with partners across [geographical location] to share resources, identify joint projects and areas for collaboration, build capacity, develop community partnerships and promote best practice.” (Leading) | |
| Have individuals’ influence, ideas and contributions been recognised and addressed? | | The importance of expressing gratitude for the effort and giving recognition of the value which PPIE adds to research is also referenced by many centres.  The NIHR Centre for Engagement & Dissemination Payment Policy has been cited as helpful to reimburse public contributors in line with this.  Centre’s asked how individual’s wanted to be recognised for their contributions.  Centres also reported providing feedback relating to their contributions. | **NIHR CED**  ARC: “PPI representatives reviewed and shaped eventual PPI strategy document. We used UK Standards for Public Involvement as framework for our objectives. Payment policies updated in line with NIHR CED.” (Learning)  **How to recognise contributions**  BRC: “[Geographical location] has a relatively small budget, and therefore does not pay for focus or advisory groups participation. The Snapshot asked respondents about reward and recognition; most wanted no reimbursement, or just expenses, 22% would like a voucher or payment, and 10% would like investment in their community.” (Listening)  **Feedback**  BRC: “All public contributors remunerated in line with NIHR guidelines, and provided with feedback on the impact of their input.” (Welcoming) | |
| **UK Standards for public Involvement – Key Lines of Enquiry/ Supportive Questions** | | **Interpretation** | **Themes, Supporting Data and links to Quality Ratings Framework**  (Links to the Quality Ratings Framework are in brackets after each quote) | |
| **Support and Learning** | | | | |
| Is there a range of support to address identified needs? | | Centres are undertaking training needs assessments and providing ongoing training, education, research toolkits and other learning opportunities to meet these gaps, building skills, knowledge and confidence.  Training and support for researchers/ staff was often co-produced with PPIE members and included: workshops/ educational sessions, PPI clinics and being sign-posted to relevant resources and guidance.  To enhance knowledge transfer researchers often wrote blogs, targeted at their peers, which focused on PPIE activities.  Inclusivity was identified as an area to address and centre’s provide support and learning on this topic.  Another identified need was better planning of PPIE activities which resulted in the development of a PPIE toolkit.  Training and support for PPIE members largely included ‘taster sessions’ via video, peer support and training for a specific project.  There seems to be a lack of training / support for PPIE members who contribute to Governance activities. | **Training needs assessments**  RDS: “Alongside our rolling programme of training, we work with our Consumer Panel to identify training and support needs, and develop sessions on reviewing lay summaries, PIF applications, and mock interview panels. We also developed training materials on the use of virtual technology.” (Leading)  **Researchers/ staff**  Co-produced training events  Research School: **“**The study involved three people with learning disabilities who co-produced and co-delivered the staff training.” (Learning)  RDS: “We work with lay members to develop and deliver sessions about the value of PPI and the support offered by the RDS to organisations regionally and nationally.   (Learning)  Workshops/ sessions  PTSRU: “We delivered the first of a series of training sessions. Eight researchers attended and interactive polls pre and post training showed increased knowledge of PPI and increased confidence delivering PPI activities. PPIE members are also invited to attend training sessions.” (Learning)  ARC: “PPI Lead collaborated with Academy to co-design/-deliver training courses for researchers to increase awareness/knowledge/confidence in involving public.” (Learning)  PPI Clinics  ARC: “Offered PPI ‘clinics’ for researchers to discuss studies/engagement challenges.” (Listening)  Using existing resources  ARC: “Signposted existing training resources from other PPI providers, e.g. (institution) Partners, (institution) BRC, NIHR CED, including training courses, online resources, reading materials.” (Welcoming)  Research School: “We updated our patient and public webpages to include a clear overview of guidance available for researchers cross-NIHR, practical advice and answers to frequently asked questions. We have included an up-to-date overview of training opportunities within the country open to researchers. These webpages are open to the wider primary care infrastructure.” (Learning)  Blogs  Research School: To enhance knowledge sharing of involvement and engagement within primary care research, the SPCR actively encourages researchers to write blogs, which are publicly available on the SPCR website. 15 blogs were published on topics such as how to involve public contributors in systematic reviews or conferences and how to engage with the public in unconventional locations such as a pub or science festival. Many blogs contain tips and tricks relevant to the primary care research community.” (Learning)  **Inclusivity:**  BRC: “We offer a range of PPI/E opportunities for contributors with different interests, needs and commitments, carefully avoiding a ‘one-size-fits-all’ approach. Our PPI/E training emphasises the need to focus on equality, diversity and inclusion (EDI) in planning activities and events, recruiting contributors and research participants and facilitating accessible engagement and participation.” (Learning)  CLARC: “The Toolkit ‘Increasing participation of Black Asian Minority Ethnic groups in Health and Social Care Research’ developed by CLAHRC EM has been adapted by ARC EM into an online module that researchers can utilise at each stage of the research process to aid them in the reach into communities for their research.” (Leading)  **PPIE Planning Tool:**  RDS: “COVID-19 helped us identify a need to support our researchers to better plan their PPIE activities. We developed a digital planner tool, which can be used by researchers to plan PPIE activities. Importantly the tool gets them to consider PPIE at different research stages, diversity and inclusion and measuring impact. We consulted with CED colleagues to ensure it would not duplicate any other tools. The planner is now in final stage of development.” (Learning)  **Training/ support for members of the public**  ‘Taster sessions’  RDS: “Some community groups wish to work with us in groups; we are developing training materials to enable this.A series of training videos, available online from July 2021, include five minute ‘taster’ sessions, and a series of one hour training events, available from September 2021, on: ‘Public Involvement and Participation’; ‘Public Engagement and Feedback’; ‘How to Identify’, ‘Approach and Engage Patients and Public.’ ”  (Learning)  **Peer support for PPIE members**  ARC: “Used ‘buddy’ system - had phone call with experienced RAP member prior to meeting to discuss how meetings worked, and to put at ease.” (Welcoming)  **Training about a specific research project**  BRC:  “Our Patients’ Voice went live in January 2021 (see section 3 and the Added Value Example) with a formal launch in February, which attracted 40 patients, carers, and members of the public. Patients and carers were involved at all design and development stages and a PPI working group has been formed. A support training session was offered in March, attracting 13 people, and it has been presented in many research meetings” (Welcoming) | |
| Have specific resources been designated to support learning and development opportunities for both the public, researchers, and staff? | | Provided financial support /bursaries for PPIE members to attend training workshops and conferences.  Unclear how staff training/ support was resourced. | **Bursaries**  Research School: “Provided 4 bursaries for public contributors to attend and participate in discussions at (conferences)” (Listening) | |
| Do the public know where to go for information and support for public involvement? | | Where infrastructure schemes have PPIE coordinators and teams locally based with their Research Teams, this forges links and promotes awareness of how to access support and learning. | **PPIE co-ordinator**  ARC: “An Involvement Learning Network would assist in support and learning; meanwhile the Involvement Coordinator continued to share links to resources and learning opportunities through a network of theme-based involvement champions and provide one-to-one support as required.” (Learning) | |
| Is there a culture of learning by doing, building on and sharing that learning for researchers, staff and the public? | | Centres reported knowledge sharing and learning through attending and presenting at various events; these events seemed to be of an academic audience. | **Learning events**  ARC: “(PPIE member) co-chaired the regional NIHR EoE PPI Collaborative sharing learning from NIHR infrastructure members across the region.” (Leading)  RDS: “We contribute to learning events across the region, including (academic) speaking at a NIHR Policy Research Funding Programme event; (academic) speaking as a Reaching Out project partner at the NIHR Applied Research Collaboration Showcase; and (staff member) promoting user led research at THIS Institute Researcher Development session on co-production. (Staff member) talked about public involvement at a virtual NIHR Fellowship event.” (Learning) | |
|  | | The National Standards were utilised by some centres as a resource to enhance support and learning. | RDS: “We recommended the use of the National Standards for practical advice on ensuring that PPI activities are inclusive.”  (Welcoming) | |
| **UK Standards for public Involvement – Key Lines of Enquiry/ Supportive Questions** | **Interpretation** | | | **Themes, Supporting Data and links to Quality Ratings Framework**  (Links to the Quality Ratings Framework are in brackets after each quote) |
| **Communications** | | | | |
| Has a communications plan been developed for involvement activities? | A number of centres co-produced a communication plan with PPIE members.  As part of the plan, PPIE members often helped to plan that study materials (e.g. consent forms and participant information sheets) were accessible to a wide range of audiences.  Within the communication plans, centres also reported involving PPIE members in the dissemination of research findings.  PPIE members generally suggested more innovative and creative methods of communicating research findings. Such methods included: illustrations, videos and graphic novels. | | | **Communication strategy**  RDS: “With input from lay members, and the wider community, we have revised communication of the details and outcomes of national and regional work. This includes a move away from often academic or clinically focussed publications or slides. We have begun work with a local illustrator who has helped us develop images to portray this information (Added Value 2).” (Learning)  **Study materials**  Research School: “(PPIE activity) included: making sure our research is relevant to (target population) (i.e. modifying our (intervention); ensuring our research materials are easily understood (i.e. PPI contributors helped write consent forms); making sure our research process is acceptable to potential participants (i.e. modifying research protocol).” (Learning)  **Videos**  Research school: “Most recently, we have seen the launch of videos involving LGBTQI+ Disable People on the findings from (programme of studies) all of which have included relevant individuals within those videos and in developing the content for them.” (Learning)  **Graphic novel**  Research School: “A (programme of study) launched a graphic novel at our annual conference in 2018 to share the findings from the study more widely.” (Learning)  **Communications and research methods**  Research School: “Research materials can be understood and that the research is acceptable to partipicants” (Learning) |
| Are the needs of different people being met through inclusive and flexible  communication methods? | Infrastructure schemes used a range of different mediums to communicate with a wide audience about PPIE opportunities; centres used both digital and printed communication strategies. Paper-based mediums were seen to ensure inclusion of individuals who may otherwise be digitally excluded.  A similar approach was also taken when communicating on-going PPIE activities to PPIE members. Strategies included innovative animations and exhibitions but also included paper-based leaflets and newsletters.  Some centres reported using frameworks (e.g. the Accessible Information Standards) to meet the communication needs of different people. The Standards set out a specific and consistent approach to identifying, recording, flagging, sharing and meeting the information and communication support needs of patients, service users, carers and parents with a disability, impairment or sensory loss.  Specific elements of communication relating to research included changing term | | | **PPIE Opportunities**  *Social media campaigns*  BRC: “Posters and an animation highlight opportunities for people to get involved in research, including as part of social media campaigns.” (Welcoming)  HPRU: “We created a twitter account to disseminate our outreach activities, promote our opportunities and establish a social media presence. We also have a dedicated section on the HPRU’s website and will continue to develop our content there.” (Welcoming)  *Newsletters*  ARC: “Opportunities for involvement are communicated to public members through the Involvement newsletter.” (Welcoming)  **On-going PPIE work**  *Animations*  BRC: “A short animation was developed to encourage people to participate (Youtube Link). This was supported by the (academics) who we have been working with on various COVID-19 PPIE and communications initiatives.” (Welcoming)  *Exhibitions*  ARC: “ARC website launched and our stakeholders are shaping this resource. Our communication strategy builds on successful engagement initiatives including: photography exhibition.” (Learning)  BRC: “We produced a series of films capturing PPIE impact for use in our digital communications.” (Learning)  *Leaflets*  PTSCR: “: - For example, within the Marginalised Groups theme we have co-produced information and promotional leaflets for involvement and engagement in (research theme)” (Learning)  **Standards for inclusive communication:**  BRC: “Our newsletter template was revised to meet Accessible Information Standards with electronic and physical copies sent out to our PPIE Network. We produced 2 large seasonal newsletters and 2 shorter monthly ones providing updates about COVID-19 information and guidance for musculoskeletal patients and our usual updates.” (Leading) |
| Are processes in place to offer, gather, act on and share feedback with the  public? | Centres generally evaluated PPIE activities against academic objectives and feedback from PPIE members.  Some centres particularly reported gathering feedback on their communication strategies.  Some centres reported that any changes to PPIE practices were fed back to PPIE members and researchers through the use of feedback loops. | | | **Evaluation processes**  ARC: “Evaluating the impact of our PPI/E is a process of: Evaluating the activity against its aims to ensure the aims have been reached; Gathering feedback from participants involved in PPI/E both participant and researcher to have a clear pathway for future improvement and shared learning” (Learning)  **Evaluating communication methods**  Research school: “Once the graphic narratives are created, wider public and practitioner networks will be engaged to evaluate the final product.” (Learning)  **Feedback loops**  HPRU: “Feedback loop will be reviewed/improved as part of strategy next period in collaboration with PPIE representatives/Steering Committee. We will pilot co-developed methods arising from this consultation.” (Leading) |
| Are you sharing your public involvement learning and achievements, good  and bad | Centres reported mobilising their learnings and achievements regarding PPIE through a variety of co-produced methods (flyers, websites, e-bulletins, social media, podcasts, events and workshops). These knowledge mobilisation strategies were for both researchers and members of the public.  A RDS is encouraging reflection upon PPIE processes and what did, and did not, work and produced a ‘practical tips’ document.  Along with mobilising learning around PPIE, centres also reported communicating their learnings of how PPIE members helped to mobilise research findings by suggesting where to disseminate the findings within the community. | | | **Knowledge mobilisation of learnings regarding PPIE practices:**  *Graphics*  ARC: “Working with the SPCR we co-designed a graphic/flyer for PPI contributors and practitioners to improve on-line meetings. This has been widely disseminated and adopted by the NIHR Academy.” (Leading)  *Websites*  RDS: “Our website and e-bulletins are kept up to date with the latest public involvement guidance and impacts and we engage with social media via our twitter account. We are working with NIHR CEDs to share lessons learned from the Reaching Out project and have co-designed a practical tips document.” (Learning)  *Podcasts*  RDS: “In partnership with CED we have recorded eight podcasts on co-production; available on our website, Spotify, SoundCloud, Anchor, as well as on the NIHR Learning and Development page and NIHR Learn. The podcasts have been listened to over 1,000 times. These podcasts were produced to help people with co-production and ensure wider accessibility to co-production issues, debates and projects. “ (Learning)  Events  RDS: We also delivered an event on co-production (attracting 25 staff from across the country), in which we shared our experiences of working co-productively at (organisation), and which was open to our lay reviewers and colleagues from other RDSs; jointly facilitated a workshop on co-production (25 attendees; including researchers and public members). (Learning)  **Knowledge mobilisation of research findings**  HPRU: “Community champions will disseminate relevant HPRU derived scientific knowledge…. This event raised awareness to disseminate charitable organisation information to patients, so they know who to contact for logistical help.” (Learning) |
| **UK Standards for public Involvement – Key Lines of Enquiry/ Supportive Questions** | **Interpretation** | | | **Themes, Supporting Data and links to Quality Ratings Framework**  (Links to the Quality Ratings Framework are in brackets after each quote) |
| **Impact** | | | | |
| Are the public involved in deciding what the assessment of impact should focus on, and the approach to take? | Infrastructure schemes used the word assessment and evaluation interchangeably. Centres described co-producing evaluation frameworks with PPIE members. Centres often reported the impact of what they described as ‘quality’ PPIE work  By gathering feedback, assessing/ evaluating PPIE activities centres are showing that they value PPIE members contributions and are creating an environment of reflection and improvement. | | | **Co-production of frameworks**  BRC:  “The Advisory Group would assist in establishing a robust measurement and evaluation framework for capturing and reporting on impact, including quantitative measures, but with an emphasis on quality, including gathering multiple perspectives and learning from what went well and where there was scope for improvement. The approach piloted in April online event, to incorporate the perspective of communities across a range of protected characteristics in addressing health inequalities and imbalances in care during the pandemic, attracted wide interest (for instance a BMJ article by EDI lead (academic) and international webinar presentation to people from 18 countries) and may be adapted for use elsewhere in England and internationally” (Leading) |
| Is it clear what information to collect to help assess impact, including who has  been involved and how? | Centres reported study-specific plans or using frameworks such as the PiiAF, KPI and ‘the cube’.  Feedback and impact logs were used to capture impact on PPIE members and research. | | | HPRU: “To facilitate the capture and reporting of impact, all PPIE activities will be annually evaluated against pre-specified indicators of success, which are captured in both the PPIE strategy and will be incorporated into the theme level PPIE plan to reflect project-level impacts. We have also developed template feedback forms for both researchers and members of the public to use following public involvement activities.” (Learning)  Research school: “Impact is assessed using study-specific plans, PIIAF, strategic KPI and academic approaches. We are developing an online service request form to automate some elements of impact assessment and quality control.” (Learning)  BRC: “The PPI evaluation template (the Cube) is now available as a resource across the HPRUC as is the recently launched online version of the ‘Cube’ (https://evaluationcube.phwe.org.uk/cube1) and the online PPI log template.” (Listening) |
| Are there processes in place to help reflect on public involvement? | Centres reported encouraging reflective practices; how they do so was not clearly reported. | | | **Reflective practices**  BRC: “We encourage researchers and public contributors to reflect on the impact and outcomes of the PPI they have been involved in through a straightforward and efficient process. We are working hard to ensure that evaluation of outcomes and impact of PPI is given appropriate attention in the research cycle, particularly in the development stages of our work.” (Welcoming) |
| Are the changes, benefits and learning resulting from public involvement acted on? | Impact case studies were used to report the changes arising from PPIE input. | | | Research School:  “We are finalising impact case stories of involvement in our studies and the difference this has made to both the research and to the individuals involved. Six have been produced and will be published on our website this month, and a further set are in development. Many of these have raised useful lessons for the future, including – for example – the role of funders in providing post-project support following completion of projects for those that have been involved and would like to continue to be involved in research. We are also working on producing a volume covering some of these experiences to be launched at an event towards the end of the year” (Listening)  BRC: “The (centre) Blog includes case studies of impact and PERC keeps a PPI activity log with outcomes.” (Listening) |
| Impact on PPIE members | Being involved in PPIE activity was reported to have a positive impact on PPIE members | | | BRC:“Making the video - and a patient being the 'face' of the training - was an enjoyable and rewarding experience. I felt like an equal in the process and felt valued and respected in terms of my input” (Leading) |
| Impact and communication methods | Through co-production of communication methods, and the sharing of PPIE activities, metrics have shown an increase in tweet impressions, twitter followers and website traffic. | | | BRC:  “We achieved 1,158,609 impressions on Twitter through sharing updates, news stories, articles and retweets about the BRC. Our website, relaunched in April 2019, has grown from 517 users in the first month to 1116 currently, an increase of 216% in two years.” (Listening) |
| Impact regionally and nationally | PPIE activity not only has impact on the PPIE members and research programmes, it also has impact on regional and national levels. | | | Research School:  “Some of the new resources have already made an impact at regional and national levels. For example:  The role descriptions of ‘RUG adviser’ will be used by partners in the (organisation) regional public involvement network, to involve public contributors to advise on the planning of public involvement in studies. The role descriptions for public members of Trials Steering Committees has been referred to in the forthcoming NIHR Charter and Terms of Reference for Trial Steering Committees.” (Leading) |
| **UK Standards for public Involvement – Key Lines of Enquiry/ Supportive Questions** | **Interpretation** | | | **Themes, Supporting Data and links to Quality Ratings Framework**  (Links to the Quality Ratings Framework are in brackets after each quote) |
| **Governance** | | | | |
| Are public voices heard, valued and respected in decision making? | PPIE members were invited to make key decisions within governance structures. Overall, public voices were heard, valued and respected on a research programme level, organisational level and regional level.  PPIE members were invited to join centre committees or board meetings, most centres also had public members as a chair or co-chair of strategy meetings.  Within the reports there was an emphasis on collective ownership regarding governance processes, including financial budgets  PPIE is also embedded within education decision-making (within the relevant centres). | | | **Research programme level**  Research School:“Public partners are involved in individual academic members’ own governance structures, contributing for example to the development of funding applications relating to data governance” (Learning)  **Organisational level**  BRC: “Our Public Partnership Advisory Group is now established in the governance structure. We co-produced the plans with a wide range of public contributors and community organisations. This included changing our structure” (Learning)  **Regional level**  “PPI representatives and/or our BRC PPI lead sit on the BRC, (organisation) and CRF steering committees, data access committee, (University) Health Partners PPI forum, (Regional Area) PPI Collaborative, steering committees of major BRC-supported research projects” (Learning)  **Collective ownership**  Research school:“PI governance processes, leadership and a central budget are in place with representatives for all members ensuring collective ownership.” (Leading)  **Education-based decision making**  Research school: “Future students: “selection panels for PhD studentships” (Leading) |
| Are public involvement plans in place that are regularly monitored, reviewed and reported on? | Centres discussed developing governance structures which are monitored and reviewed within board meetings. The review of such processes is enabled by integrating PPIE as an item on board meeting’s agendas.  Regular meetings between centre PPIE teams and project managers to review PPIE activities; it was unclear if PPIE members were involved in these meetings. | | | **Board meetings:**  PSTRU: “Lay Leaders continue to contribute to the Executive and Advisory Boards and have developed a governance process to support involvement in the meetings, including a preparatory meeting with the programme manager and submission of a PPIE paper outlining the Lay Leaders collective ideas, challenges and questions. PPIE is a standing item at all meetings.” (Leading)  **Regular meetings**  HPRU: “The PPI team meet monthly with the HPRU project manager to review and develop PPI work plans. We have also developed a working group bringing together PPI, Knowledge Mobilisation, Comms and training to ensure the joined up development/ delivery of our strategy.” (Learning) |
| Is there visible and accountable responsibility for public involvement  throughout the organisation? | Centres usually reported having PPIE leads and academic advisors within their PPIE infrastructure.  PPIE leads are individuals who are responsible and accountable for leading PPIE activities within a research project. An Academic PPIE advisor has expertise which is specifically related to public involvement.  Another role was that of a PPIE champion. PPIE champions are patients or members of the public whose responsibility is to provide a link between NHS trusts and Clinical Research Networks. PPIE champions were embedded into governance structures. | | | **PPIE leads and academic advisors**  ARC: “PPIE steering group being formed to take forward strategy and will meet in next period. This will be led by PPIE Lead and will include ARC Academic PPIE advisor (who has particular expertise in community involvement), PPIE representatives and ARC researchers. Core PPIE infrastructure comprises: Research Advisory Panel (RAP) of local patient/public representatives, who meet approx. monthly and provide advice/feedback to individuals/study teams at all stages of research”. (Learning)  **PPI Champions:**  ARC: “ARC governance structure includes PPI Champions embedded in each theme and two Public Members on the Strategy Board.” (Learning) |
| Are realistic resources (including money, staff, time) allocated for public  involvement? | Centres ensured that PPIE activity within governance structures was funded, thus adding to sustainability of such activities.  Centres allocated staff time to specifically focus on PPIE activities.” | | | **Funding**  HPRU: “The HPRU has funded, and continues to fund, the initiation and long-term support of a DOTW patient advisory panel.” (Welcoming)  **Staff allocation**  HPRU: Patient and Public Involvement and Engagement (PPIE) is led (academic lead) at 10% fte.” (Leading) |
| Is the privacy of personal information protected by collecting and using it in a suitable way? | Centres did not clearly report governance of personal information and how such information would be used. Perhaps a formal way to collect and develop of a guide for using PPIE members’ personal information can be developed? | | |  |
