# Supplementary material for: Using the UK standards for public involvement to evaluate the public involvement sections of annual reports from NIHR managed research centres
Source: Res Involv Engagem. 2023 Nov 30;9:109. doi: 10.1186/s40900-023-00517-3 (PMC10688454; doi:10.1186/s40900-023-00517-3)
Supplement: Supplementary file 3 — Additional file 3. The GRIPP 2 Short Form. [file 40900_2023_517_MOESM3_ESM.docx]

Additional File Three: The GRIPP2 Short Form

| Section and topic | Item |
| --- | --- |
| 1: Aim  Report the aim | To integrate PPIE into four key stages of the research process:  1) When developing the research questions,  2) When developing the methods,  3) When interpreting the findings,  4) When developing the recommendations. |
| 2: Methods  Provide a clear description of the methods used for patient and public involvement (PPI) in the study | The researchers convened a PPIE group (two members of Keele Medical Schools Research and User Group and an individual aligned to the School of Allied Health, Keele University) specifically for this study.  AM met with the PPIE members on three occasions via Microsoft Teams.  Within the first meeting the research questions, UK standards and Quality Insights Framework were discussed.  Within the second meeting the methods were further discussed and the initial findings were presented.  The third meeting focused on the findings and developing recommendations. |
| 3: Results  Outcomes—Report the results of PPI in the study, including both positive and negative outcomes | Whilst the scope of the research (e.g. to review PPIE sections of annual reports) had already been developed and commissioned by the NIHR, the researchers worked with the PPIE team to develop the specific research questions. When developing the methods, the researchers discussed the analysis framework and co-produced the working definitions of each UK standard with the PPIE group. The PPIE group also suggested that using the ‘leading’ and ‘learning’ domains from the Quality Ratings Framework would be an appropriate way to recognise good PPIE practice.  PPIE members were also invited to share their perspectives on the research findings. The PPIE group where shown anonymised data from reports. The quotes were chosen to illuminate a ‘supporting question/ key line of enquiry’ within the Data Extraction Table. The PPIE members provided their interpretation of the data which mostly agreed with researchers’ interpretations.  The recommendations were also developed through researchers, wider stakeholders and the PPIE group working together. |
| 4: Discussion  Outcomes—Comment on the extent to which PPI influenced the study overall. Describe positive and negative effects | Whilst PPIE has been integral to this study, PPIE members did feel that they could not greatly influence the research questions as these had already been set out by the NIHR.  PPIE members thought the use of the UK Standards and Quality Insights Framework was appropriate, especially as both frameworks had been co-produced by academics and public members.  When developing the recommendations, PPIE members specifically wanted recommendations focusing on what they could contribute (e.g. continuing to promote the use of the UK Standards). |
| 5: Reflections  Critical perspective—Comment critically on the study, reflecting on the things that went well and those that did not, so others can learn from this experience | Whilst PPI has been integral to this study, PPIE members could not influence the topic of the research questions as these had already been set out by the NIHR. There needs to be clarity regarding the roles of public contributors within a study and what they can, and cannot, influence and that, ideally, public contributors should be involved in the conception of a study.  As this was a six month evaluation, it was difficult for the research team to work with PPIE members to truly share power and responsibility from the start to the end of the project as this would have needed a much longer time-period.  PPIE members’ perspectives on the data did not differ from the interpretation presented to them. Although this arguably enhances the trustworthiness of the findings, AM thought that it could have also been due to power dynamics. Public contributors may not have challenged the interpretation presented to them due to the perception that they did not have enough power, or experience in qualitative data analysis methods, to influence the analysis.  Researchers and PPIE members met to discuss PPIE impacts. The group noted that the PPIE members involved in this study were all experienced and knew of the UK Standards and Quality Insights Framework; the group questioned if less experienced PPIE members would have been able to contribute.  All PPIE members were older Caucasian males. In the future, researchers will try to seek a more diverse group. The building of relationships with under-served communities takes time which needs to be factored into project timelines. Due to time and capacity restraints, we could not fully involve Keele Medical School’s Race Equality Ambassador. In future, we endeavor to include this individual to enhance involvement with those from under-served communities. |
